# Age, period, cohort effects in trends of depressive symptoms among middle-aged and older Chinese adults

**DOI:** 10.3389/fpubh.2024.1383512

**Published:** 2024-07-31

**Authors:** Xiaoqian Hu, Wenxue Jin, Junlei Wang, Hengjin Dong

**Affiliations:** ^1^School of Politics and Public Administration, Qingdao University, Qingdao, China; ^2^Center for Health Policy Studies, School of Public Health, Zhejiang University School of Medicine, Hangzhou, China; ^3^The Fourth Affiliated Hospital, Zhejiang University School of Medicine, Hangzhou, China

**Keywords:** age-period-cohort, depression, China, urban–rural disparity, life course

## Abstract

**Objectives:**

To investigate the effects of age, period, and cohort on the trends of depression; and to examine the influence of these three temporal effects on residential disparities in depression.

**Methods:**

Using data from the China Health and Retirement Longitudinal Study (CHARLS) during 2011 to 2020, involving 77,703 respondents aged 45 years old and above. The measurement of depressive symptoms was the score of 10-question version of the Center for Epidemiologic Studies Depression Scale (CES-D 10). The hierarchical age-period-cohort cross-classified random effects models were conducted to examine trends in depressive symptoms related to age, period and cohort.

**Results:**

CES-D scores increased with age and slightly decreased at older age. The cohort trends mostly increased except for a downward trend among those born in 1950s. As for the period effect, CES-D scores decreased gradually from 2011 to 2013 followed by a upward trend. Rural residents were associated with higher level of depression than those live in urban area. These residence gaps in depression enlarged before the age of 80, and then narrowed. The urban–rural disparities in CES-D scores gradually diminished across cohorts, while the corresponding period-based change in urban–rural gaps was not significant.

**Conclusion:**

When age, period, cohort factors are considered, the age effects on depression dominated, and the period and cohort variations were relatively small. The residence disparities in depression reduced with successive cohorts, more attention should be paid to the worsening depression condition of younger cohorts in urban areas.

## 1 Introduction

Depression, a significant mental health concern, has attained global significance as a public health challenge. The Global Burden of Disease Study (GBD) 2019 reports that approximately 280 million individuals worldwide are affected by depression (1). Depression is expected to be the leading cause of the global burden of disease by 2030 (2). Middle-age and older adults appear to be more vulnerable to depressive disorders (3). Older people with depression are at elevated risks for disabilities, chronic diseases and mortality, causing serious social and economic burdens (4–6). In China, more than 50.06 million people lived with depressive disorders in 2019, accounting for 17.8% of global cases (1). With the accelerated trend of population ageing in China (7), more attention to depression in later adulthood is urgently needed.

Understanding variations in late-life depression is becoming critically important, especially for the development of social security, health care delivery, and long-term care policies. Previous studies demonstrated that depression changes were related to three unique temporal factors: age, time period and cohort (8). First, as for the age effect, along with the biological and social ageing process, key predictors of depression such as social isolation and poor health are more prevalent in older age (9). Prior studies identified that depressive symptoms tend to increase as people age (10–12). Second, the period effect, referring to a range of environmental, social or economic factors during a particular period might result in depression variations. Growing economic uncertainty, public health emergencies, and health promotion programs may have effects on depressive symptoms (12, 13). Third, depression could be affected by cohort effects, reflecting the unique experiences or exposures throught the life course of distinct generations (14, 15). For example, previous studies demonstrated that prenatal exposure to the famines had proven to exert a long-lasting negative impact on mental health in later life (16–18).

Considering that age, period and cohort (APC) had distinct effects on depressive symptoms, numerous studies explored the time trends in depression (19–22), while few of them simultaneously adjusted for the age, period and cohort effects. However, failure to isolate APC trends may lead to substantial bias (23). Existing researches exploring APC trends in depression simultaneously mostly focused on Western countries with conflicting results. A Germany study identified a U-shaped cohort trend and a decreasing period trend on depression (11). Another study analyzed depression variations among older Canadians and demonstrated an declined trend in depressive symptoms with age, a non-linear increasing trend across successive birth cohorts and a non-significant fluctuated trend with period (24). To our knowledge, few studies simultaneously investigated the APC trends in depression among Chinese older population. A Chinese study focused on incidence trends of major depressive disorder using the GBD 2017 data, and reported a basically increasing trend in incidence of major depressive disorder (MDD) with age, a largely decline over time, and an inverted U-shaped pattern across cohorts (25). While this study employed estimated data and examined the impact of age on the incidence of MDD by 5-year age intervals (e.g., incidence of MDD for 60-64-year-old age group) rather than 1-year age intervals, thus the estimation of APC effects could be further evaluated in much detail with population-based data. Meanwhile, China has undergone a tremendous societal transition and a remarkable economic expansion, Chinese population have been exposed to unique and powerful social forces (26). It is anticipated that these societal changes would have distinct impacts on the health of populations in different periods and birth cohorts, which may differ from what has been observed in western countries. However, large-scales studies on the APC trends in depression among the Chinese population are scarce and are looking worthy of further investigation.

Urban–rural gaps in depression had attracted plenty of attention in the worldwide (27, 28). In developed countries, studies have revealed a higher prevalence of depressive symptoms among urban residents compared to their rural counterparts (29). While several studies conducted in China consistently demonstrated that rural residents exhibited higher levels of depressive symptoms than those residing in urban areas (30, 31), which probably results from the urban–rural disparities in socio-economic factors, health systems, social support and social participation for a long history (32, 33). Given that rapid urbanization process in China, the urban–rural disparities in depression may also change. However, few studies have examined the effects of age, period and cohort on residence differences in depression using comprehensive temporal models. Wu identified that the urban–rural disparities widen among younger Chinese older adults, and narrowed after the age of 80 (34). While this study did not identify time period and birth cohort trends. Further investigation is needed to explicate the impact of APC on the trends of urban–rural gaps in depressive symptoms.

This study therefore has two main objectives. Firstly, to investigate the effects of age, period, and cohort on the trends of depression; Secondly, to assess the influence of these three temporal effects on residential disparities in depression. We utilized data from the China Health and Retirement Longitudinal Study (CHARLS) and adopted hierarchical APC cross-classified random effects models (HAPC-CCREM) to achieve these objectives.

## 2 Materials and methods

### 2.1 Data source

We used data from the CHARLS, which was a panel survey of Chinese residents aged 45 and above. The main goal of CHARLS is to provide a high quality nationally representative sample of Chinese residents’ information to serve the needs of scientific research on health, economic position, and quality of life as people age. More details about CHARLS data was introduced by Zhao previously (35). CHARLS used a stratified (by *per capita* GDP of urban districts and rural counties) multi-stage (county-level, neighborhood-level, household-level, and respondent-level) proportionate to population size (PPS) random sampling method to control the quality and representativeness of the samples (details about sampling design of the CHARLS were shown in https://charls.pku.edu.cn/en/About/Sample.htm). The CHARLS National Baseline Survey was launched in 2011, covering more than 17,000 respondents from 150 counties and 450 communities (villages) of 28 provinces (autonomous regions and municipalities) in China. Four follow-up surveys with replacements for deceased samples were conducted in 2013, 2015, 2018, and 2020. The survey encompassed a comprehensive collection of data, including demographics, income and assets, health status, cognitive abilities, family structure, healthcare utilization and costs, job status and history, insurance and biomarker. The CHARLS had been approved by Biomedical Ethics Review Committee of Peking University.

### 2.2 Study samples

The CHARLS contains both nationally representative cross-sectional and longitudinal samples. Until now, CHARLS conducted about ten years including middle-aged and older Chinese people across successive cohorts, which provided the possibility for exploring the effects of age, period, cohort on depression trends. Five-wave data (2011, 2013, 2015, 2018, and 2020) was used in our analysis. Firstly, 92,733 samples aged 45 and over were involved in our analysis. Then, 6 respondents older than 105 years old were excluded due to self-reported age after 105 years is not reliable (36). Finally, after exclusion of samples with missing values in the measures of depression, we obtained 77,703 respondents as the final sample size. Detailed description of the sample selection is shown in Supplementary Figure S1. In order to reduce the potential bias of excluding the missing data, we performed multiple imputation on CES-D scores and results were not substantially different from the analyses without imputation (details were shown in Supplementary Tables S2, S3 and Supplementary Figures S2, S3).

### 2.3 Variables

#### 2.3.1 Depressive symptoms

The 10-question version of the Center for Epidemiologic Studies Depression Scale (CES-D 10) was used in CHARLS questionnaires to measure depressive symptoms of the respondents (details were shown in Supplementary Table S1). The CES-D 10 is a self-report scale designed by Radloff to assess the severity of depressive symptoms among the general population (37), which was demonstrated by numerous studies to have good reliability and validity in different cultures worldwide (38–40). The respondents were asked to report the frequency of 10 depressive symptoms in the past week on a four-point scale, including rarely (<1 day), some days (1–2 days), occasionally (3–4 days), and most of the time (5–7 days). For eight negative symptoms (such as felt lonely), these four options were assigned as 0, 1, 2, and 3 in turn. For two positive symptoms (such as felt hopeful), the coded reversed as 3, 2, 1, and 0. The sum of the 10 items were CES-D scores in our research. It ranged from 0 to 30, with high value referring to serve depression (Chronbach’s alpha = 0.815) (41).

#### 2.3.2 Age, period, and cohort

Age, in other words chronological age, was years since birth, ranging from 45 to 105 in our analyses. The age variable was transformed by subtracting the value from the grand mean and further being divided by ten for ease of interpretation of the intercept values (8). Period referring to the year in which the interview conducted, which included 2011, 2013, 2015, 2018 and 2020. The cohort classification was based on the year of birth of the participants. In order to ensure a sufficient sample size, individuals born before 1938 or after 1969 were grouped separately (42). Subsequently, the remaining birth cohorts were grouped into three-year intervals. The cohort variable was treated as a continuous measure (43), with the oldest cohort coded 1 and the youngest cohort coded 12.

#### 2.3.3 Covariates

Residence, the main stratification factor, indicated the respondents’ living region (urban or rural) at the survey. It was categorized based on the classification established by the National Bureau of Statistics of the People’s Republic of China. Specifically, a code of 0 denoted the household was in an urban area, while a code of 1 denoted the household was in a rural region.

Due to depressive symptoms were affected by many factors, we included several demographic characters, socioeconomic status (SES) and health status as covariates (9, 41). Demographic characters included sex (male, and female), co-residence (alone, and with others) and marital status (with spouse, and without spouse). Respondents who were married with spouse and cohabited were coded as “with spouse,” those divorced, widowed, and never married were coded as “without spouse.” SES included education level (illiterate, primary, secondary or above) and working status (not currently working, and currently working). Education level identified the highest level of education that the respondent completed. Working status indicated whether the respondent engaged in any work in the past year. Respondents’ health status was measured by activities of daily living (ADL) disability, which was the number of 6-item (bathing, dressing, eating, getting in/out of bed, using the toilet, and controlling urination) that participants cannot perform independently.

### 2.4 Statistical methods

We applied a descriptive statistics to summarize the depressive symptoms and other confounding factors for every wave. Thereafter, we fit hierarchical age-period-cohort cross-classified random effect regression models (HAPC-CCREM) to examine the age, period, and cohort effects on depression simultaneously (8). HAPC-CCREM used two-level model to estimate APC effects, where age was assumed to have fixed effect in the first level, and periods and cohorts were estimated as random effects in the second level (44). In order to solve the classical APC identification problem, the HAPC model used different temporal grouping for the age, period, and cohort components. On the one hand, birth cohorts were defined by 3-year intervals to break the linear dependence among three temporal dimensions. On the other hand, the nonlinear transformation method recommended applying a parametric nonlinear transformation (e.g., polynomials) to at least one of the APC dimensions in order to break their linear relationships. Coupled with prior researches highlighting the non-linear effect of age on mental health (44–46), we therefore conducted models of CES-D scores as a quadratic function of age.

In the development of each model, CES-D scores were regressed on age in linear and squared terms and other confounding variables were included as needed. The coefficients of the cohort period, and residence were permitted to have random effects (44), thereby enabling a comprehensive exploration of the period-based and cohort-based variations in the urban–rural disparities of depressive symptoms.

The model was specified in the following form:

Level-1 Model:


$$\begin{array}{l} CES-D_{ijk}=\beta_{0jk}+\beta_{1}Age_{ijk}+\beta_{2}Age_{ijk}^{2}+\beta_{3jk}Res_{ijk}+\\ \;\overset{P}{\underset{p=4}{\sum }}\beta_{p}X_{\mathrm{pijk}}+e_{ijk},e_{ijk}\sim N\left(0,\sigma^{2}\right) \end{array}$$

where $CES-D_{ijk}$ describes scores of CES-D for respondent *i* (for *i* = 1, 2, …, $n_{jk}$ ) within period *j* (for *j* = 1, 2, …, 5) and cohort *k* (for *k* = 1, 2, …, 11); $\beta_{0jk}$ is the intercept indicating the average CES-D score for the reference group at the mean age interviewed within a specific period *j* and cohort *k*; Age and Age^2^ denote age and age-squared, respectively, where Age is centered around its grand mean (divided by 10); $\beta_{1}$ and $\beta_{2}$ denote the fixed coefficients for age; Res denotes residence; $\beta_{3jk}$ denotes the random coefficients for residence; $X_{p}$ represents other individual-level variables, including interaction between age and residence, to explore how the urban–rural disparity in depression varied with age and confounding factors. $\beta_{p}$ denotes fixed coefficients for covariates; *P* is the maximum number of covariates included; $e_{ijk}$ represents an individual-level random error term.

Level-2 Model:


$$\beta_{0jk}=\gamma_{0}+u_{0j}+v_{0k}$$

$$\beta_{3jk}=\gamma_{3}+u_{3j}+v_{3k}$$

$\beta_{0jk}$ denotes a random intercept, which specifies that the overall mean across from different periods and cohorts. $\gamma_{0}$ represents the overall mean or intercept; $u_{0j}$ is the overall period effect, which is the average of the residual random coefficients for period *j* across all cohorts; $v_{0k}$ is the overall cohort effect, which is the average of the residual random coefficients for cohort *k* across all periods. $\beta_{3jk}$ denotes the random coefficients for residence; $\gamma_{3}$ represents the fixed effects of residence. To investigate whether urban–rural disparities in CES-D scores differ across periods or cohorts, we specify that coefficients have period effects ( $u_{3j}$ ), and cohort effects ( $v_{3k}$ ). In our analysis, the random variance components associated with the intercept and coefficients, which are attributed to specific periods and cohorts, are assumed to have multivariate normal distributions (44).

Utilizing a combination of two-level models, we formulated six distinct analyses to investigate the comprehensive impacts of age, period, and cohort on depression trends, as well as the change of urban–rural disparities in depression with age, period and cohort. Model 1 explored the net effects of APC on CES-D scores using age and age-squared as fixed effects and period and cohort as random effects. From Model 2 to Model 4, residence, interaction between age and residence, confounding variables were added successively to explore their potential effects on CES-D scores. In Model 5, the random effect of residence coefficient was added to explore the period-based and cohort-based trends of urban–rural disparities in CES-D scores. Model 6 added covariates on the basis of Model 5 to form a full model. Estimated CES-D scores were displayed in figures from selected models to illustrated the trends. Analyses were carried out utilizing SAS PROC MIXED (18). Bayesian Information Criterion (BIC) served as a metric to compare the fitness among models, the smaller BIC value indicating the better model fit (36). All analyses were weighted using individual sample weights, adjusted for non-response.

## 3 Results

### 3.1 Basic characteristics of samples

Table 1 showed the basic characteristics of samples from 2011 to 2020. The total number of participants were 77,703, with an average age of 60.07 years old. Nearly 60% of the individuals live in rural area. Most of the respondents lived with others and married. Around one-fifth respondents were illiterate. The mean CES-D score for all participants was 8.25, with a range of 7.83 to 8.64 across the five surveys.

**Table 1:** Basic characteristics of samples in five surveys.

Variables	ALL (*N* = 77,703)	2011 (*N* = 14,413)	2013 (*N* = 14,922)	2015 (*N* = 16,818)	2018 (*N* = 15,675)	2020 (*N* = 15,875)
Age	60.07 ± 9.43	58.91 ± 9.50	59.29 ± 9.33	59.41 ± 9.52	60.54 ± 9.29	62.09 ± 9.15
Sex						
Male	37,894 (48.8)	6,951 (48.2)	7,296 (48.9)	8,357 (49.7)	7,678 (49.0)	7,612 (47.9)
Female	39,809 (51.2)	7,462 (51.8)	7,626 (51.1)	8,461 (50.3)	7,997 (51.0)	8,263 (52.1)
Residence						
Urban	31,256 (40.2)	5,799 (40.2)	5,977 (40.1)	6,740 (40.1)	6,382 (40.7)	6,358 (40.1)
Rural	46,447 (59.8)	8,614 (59.8)	8,945 (59.9)	10,078 (59.9)	9,293 (59.3)	9,517 (59.9)
Co-residence						
Alone	4,750 (6.1)	838 (5.8)	665 (4.5)	770 (4.6)	1,237 (7.9)	1,240 (7.8)
With others	72,953 (93.9)	13,575 (94.2)	14,257 (95.5)	16,048 (95.4)	14,438 (92.1)	14,635 (92.2)
Marital status						
With spouse	68,063 (87.6)	12,625 (87.6)	13,192 (88.4)	14,822 (88.1)	13,730 (87.6)	13,694 (86.3)
Without spouse	9,640 (12.4)	1788 (12.4)	1730 (11.6)	1996 (11.9)	1945 (12.4)	2,181 (13.7)
Education						
Illiterate	17,537 (22.6)	3,839 (26.6)	3,583 (24.0)	3,758 (22.3)	3,144 (20.1)	3,213 (20.2)
primary	34,019 (43.8)	5,706 (39.6)	6,042 (40.5)	7,599 (45.2)	7,325 (46.7)	7,347 (46.3)
Secondary or above	26,144 (33.6)	4,868 (33.8)	5,297 (35.5)	5,458 (32.5)	5,206 (33.2)	5,315 (33.5)
Missing^^^	3 (<0.1)	–	–	3 (<0.1)	–	–
Working status						
Not currently working	25,712 (33.4)	5,281 (37.2)	4,789 (32.6)	5,336 (32.3)	5,159 (33.0)	5,147 (32.4)
Currently working	51,218 (66.6)	8,911 (62.8)	9,907 (67.4)	11,176 (67.7)	10,497 (67.0)	10,727 (67.6)
Missing	773 (1.0)	221 (1.5)	226 (1.5)	306 (1.8)	19 (0.1)	1 (<0.1)
ADL limitations	0.34 ± 0.91	0.33 ± 0.95	0.29 ± 0.83	0.35 ± 0.92	0.32 ± 0.89	0.39 ± 0.97
CES-D score	8.25 ± 6.32	8.44 ± 6.37	7.83 ± 5.78	7.92 ± 6.39	8.43 ± 6.49	8.64 ± 6.46

CES-D, the center for epidemiologic studies depression scale. ADL, activities of daily living.Data are presented as mean ± standard deviation or *n* (%). ^^^ Missing data were excluded from other percentage calculation.

### 3.2 Overall age-period-cohort trends in CES-D scores

**Figure 1:**
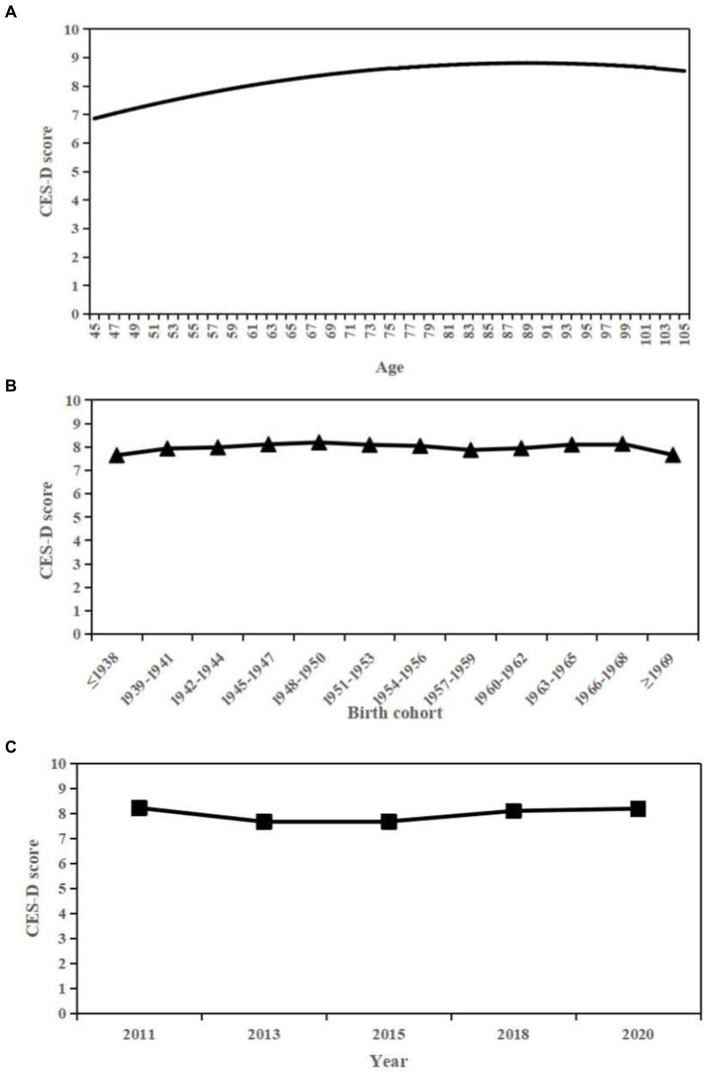
Overall age, period, and cohort effects on CES-D scores. **(A)** Age. **(B)** Cohort. **(C)** Period.

Table 2 showed the estimates of fixed effects of all individual-level covariates and random-effect variance components. When period and cohort effects were taken into consideration, the age effect on CES-D scores was curvilinear (coef. For age = 0.585, *p* < 0.001; coef. For age^2^ = −0.103, *p* < 0.001). That is CES-D scores increased with age before 90 years old then slightly decreased (Figure 1A). Relative to the age effect, the period and cohort effect were relatively small. The cohort effects were more relevant to the explanation of changes in CES-D scores than period effects (coef. for period = 0.079, *p* = 0.086; coef. for cohort = 0.041, *p* = 0.042). As for cohort effects, CES-D scores increased in the older cohorts born before 1950, followed by a slight downward trend for those born between 1950 and 1959, while increased again thereafter (Figure 1B). In terms of period effects, the estimated CES-D scores decreased from 2011 to 2013, followed by a increasing trend (Figure 1C). Other covariates suggested that female and less educated people tended to have more depressive symptoms. Those lived with spouse had a relatively lower CES-D scores than counterparts who were not married. Physically healthier individuals had fewer depressive symptoms either.

**Table 2:** Hierarchical age-period-cohort cross-classified random-effects model estimates of CES-D scores.

	Model 1		Model 2		Model 3		Model 4		Model 5		Model 6	
	Coefficient	SE	Coefficient	SE	Coefficient	SE	Coefficient	SE	Coefficient	SE	Coefficient	SE
Fixed effects												
Intercept	7.977^***^	0.144	6.937^***^	0.145	6.870^***^	0.147	6.670^***^	0.195	6.896^***^	0.139	6.677^***^	0.186
Age	0.585^***^	0.060	0.581^***^	0.049	0.430^***^	0.057	−0.091	0.071	0.507^***^	0.071	−0.077	0.077
Age^2^	−0.103^***^	0.031	−0.094^***^	0.029	−0.018	0.035	−0.090^**^	0.035	−0.061^*^	0.031	−0.093^*^	0.038
Residence (urban = 0)			2.080^***^	0.045	2.205^***^	0.058	1.535^***^	0.058	2.116^***^	0.139	1.522^***^	0.115
Age* Residence					0.303^***^	0.051	0.144^***^	0.049			0.115	0.075
Age^2^ * Residence					−0.145^***^	0.041	−0.151^***^	0.039			−0.130^***^	0.049
Sex (male = 0)							1.457^***^	0.045			1.454^***^	0.045
Education (illiterate = 0)												
Primary							−0.500^***^	0.061			−0.504^***^	0.061
Secondary or above							−1.616^***^	0.066			−1.622^***^	0.066
Marital status (with spouse = 0)							1.280^***^	0.079			1.280^***^	0.079
Co-residence (alone = 0)							−0.064	0.105			−0.067	0.105
Working status (no = 0)							−0.036	0.050			−0.036	0.050
ADL limitations							2.072^***^	0.025			2.072^***^	0.025
Variance components												
Period												
Intercept	0.079	0.058	0.086	0.062	0.086	0.063	0.076	0.056	0.060	0.046	0.051	0.040
Residence									0.037	0.034	0.026	0.025
Cohort												
Intercept	0.041^*^	0.024	0.023	0.015	0.025	0.016	0.053^*^	0.029	0.062^*^	0.033	0.071^*^	0.039
Residence									0.116^*^	0.059	0.041	0.028
Model fit												
BIC	493220.2		491077.6		491050.2		470347.0		491027.4		470327.2	

CES-D, the center for epidemiologic studies depression scale. SE, standard error. ADL, activities of daily living. BIC, Bayesian Information Criterion. * *p* ≤ 0.05; ** *p* ≤ 0.01; *** *p* ≤ 0.001.

### 3.3 Age-period-cohort trends of urban–rural disparities in CES-D scores

**Figure 2:**
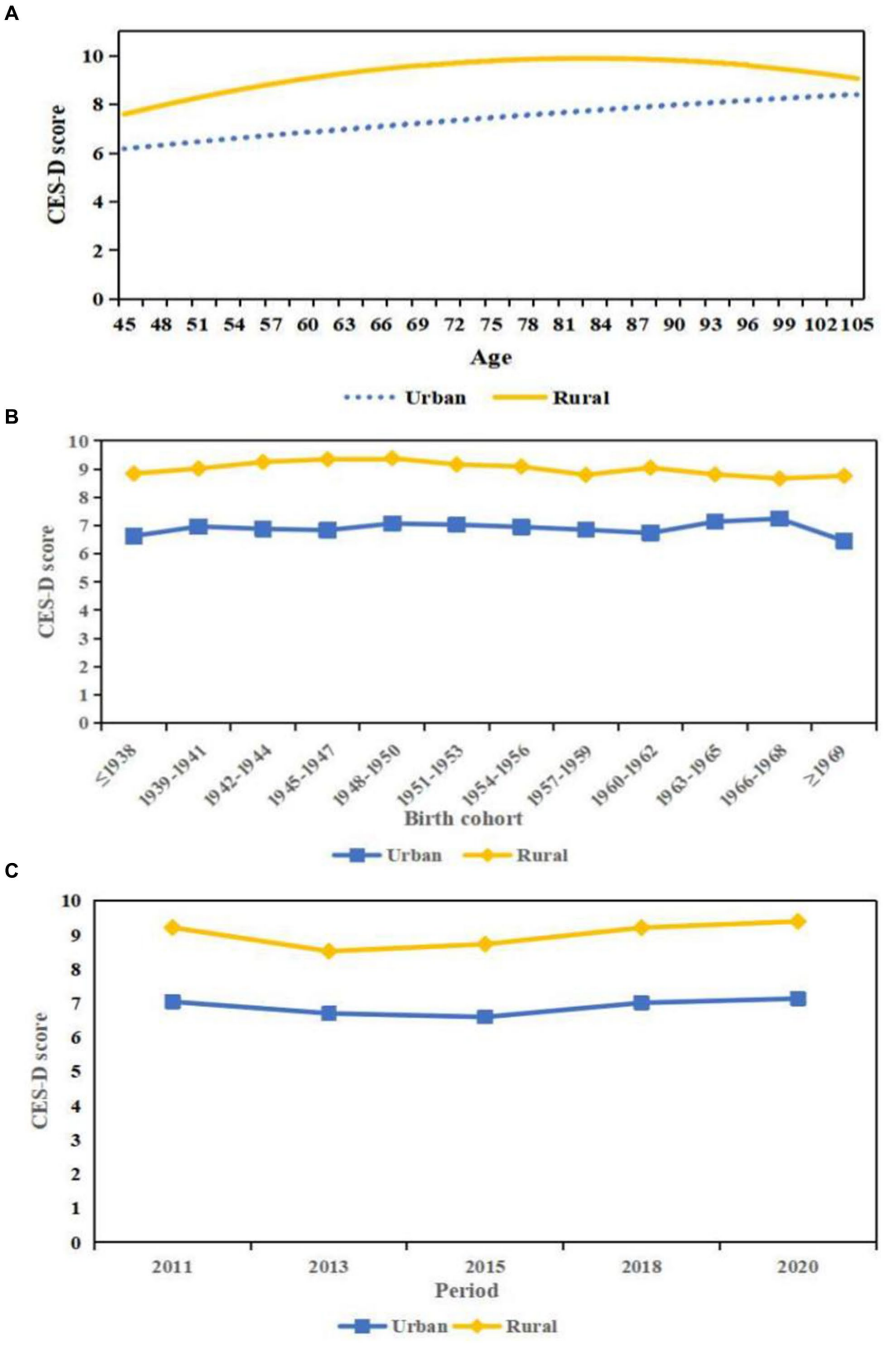
Predicted age, period, and cohort trends in the urban–rural disparity in CES-D scores. **(A)** Age. **(B)** Cohort. **(C)** Period.

Model 2 indicated that rural residence on average had significant higher CES-D scores relative to those live in urban area (coef. = 2.080, *p* < 0.001), when period and cohort effects were taken into account. For the interaction terms in Model 3 indicated that the urban–rural differentials in CES-D scores varied significantly as people get older (coef. for age = 0.303, *p* < 0.001; coef. for age^2^ = 0.145, *p* < 0.001). Urban residents experienced a continuous upward trend in CES-D scores with age. The CES-D scores of rural residents increased rapidly before 80, and then decreased gradually with age. As a result, urban residents’ advantage on CES-D scores over rural counterpart widened and then narrowed (Figure 2A).

When major correlates of depression were held constant in Model 4, the residence effects in CES-D scores still persisted but decreased in size, indicating that some of the effect of urban–rural gaps were mediated by covariates. The interaction effect of residence with age remained significant when confounding variables were controlled.

Model 5 demonstrated that there seemed to be significant cohort changes in levels of depression and urban–rural inequalities net of age effects, period changes, and other covariates. Figure 2B illustrated that the urban–rural disparities in CES-D scores gradually diminished across cohorts. Specifically, urban and rural residents experienced a similar upward trend in CES-D scores for those born before 1950. For those rural residents born after 1950, the CES-D scores decreased with successive cohorts. While the estimated CES-D scores of urban residents were relatively stable for 1950s, then increased for those born between 1960 and 1968, which leading to a reduction in the urban–rural gaps in the CES-D scores for those younger cohorts. The insignificant coefficients of period shown in level 2 indicated that the urban–rural disparities were constant during the last 10 years when age effects, cohort effects and other factors were controlled (Figure 2C).

## 4 Discussion

Using a large, nationally longitudinal dataset of middle-aged and older Chinese people, we applied HAPC-CCREM to explore age, period, cohort trends of depressive symptoms, simultaneously. CES-D scores increased with age and slightly decelerated at older age. The cohort trends largely increased except for a downward among those born in 1950s. Rural residents were associated with higher CES-D scores than those live in urban area. These residence gaps in CES-D scores enlarged before the age of 80, and then gradually narrowed. The urban–rural disparities in CES-D scores diminished across cohorts, while the corresponding period-based change in urban–rural gaps was not significant.

These findings of the distinct effects of age, period and cohort on depression trends among Chinese middle-aged and older adults indicated the importance to test APC effects simultaneously in studies of health changes. In consistent with prior studies (21, 44), our findings indicated depressive symptoms increased with age at a decreasing growth rate. Specifically, age-based depression level demonstrated a upward trend before the age of 90, then followed by a slight downward trend. The age effects were strong and independent of period and cohort effects. With age, people are exposed to more risk factors related to depression, including physiological and social factors. Regarding physiological aspects, older people are more likely to suffer from physical illnesses and disability, resulting in an increased risk of depression (31, 47). Social factors such as retirement and widowhood often separated older adults from mainstream society and social network, leading people in later adulthood to be more vulnerable to depressive symptoms (48). Similarly, an American study used more than 30 years of depressive symptom assessments from the Baltimore Longitudinal Study of Aging and observed that the CES-D scores increased at the older adulthood (49). While the slight decreasing trends of depressive symptoms at advanced age we found may partly be explained by selective survival effect (44). Those nonagenarians and centenarians who could survive to advanced ages suffering possible hardships in early life stage were likely to be more robust and more optimistic towards life (50).

Regarding cohort effects, the level of depression increased in the older cohorts born before 1950, followed by a downward trend for those born between 1950 and 1959, while increased again among the younger cohorts. These cohort trends in depression might indicate the potential impacts of societal shifts and individual life experiences on depression (21, 51). The increasing trend of depression observed among older cohorts before 1950 may due to the long-lasting damage affected by the social and political disturbances, such as the War of Resistance (1931–1945) and the Chinese Civil War (1927–1949). Researches demonstrated that early life adversity would have profound effects on mental health during the whole life (52, 53). The highest CES-D scores were shown in the 1948–1950 cohort, who experienced war and chaos at birth, and the Great Chinese Famine (1959–1961) in childhood. At their youth, they may lose opportunities to receive education further during the Cultural Revolution (1966–1976). These historical events they suffered through the lifespan might be risk factors for depression (25). After the establishment of the People’s Republic of China in 1949, a downward trend for those born between 1950 and 1959 were shown. The decreasing trend in depression among 1950s cohorts was in line with another Chinese study, which identified the incidence of major depressive disorder began to decline from those born after 1950 (25). Unlike the older cohorts, their childhood was in a relative peaceful condition. Meanwhile, the Anti-illiteracy Movements and mass public health campaigns were launched since the 1950s, which might play an important role in the improvement in mental health (43, 51). The increasing trend in depression among younger cohorts were consistent with prior studies (11, 24, 25). The possible reason may be that the younger cohorts might be exposed to increasing depression risks (11, 22). The growing economic uncertainly, accelerated pace of modern life, along with increasingly stress might result in a higher possibility of anxiety, disappointment, and distress, which may increase the levels of depression among younger cohorts (54, 55). Moreover, the rise of individualistic and consumer culture emphasizing extrinsic values (like money and fame) might also lend some explanation for the observed mostly rising trends in depression (54). The period effects on overall depression net of age and cohort effects were insignificant, indicating the depression variations mainly explained by age and cohort effects from 2011 to 2020. While this result is different from some previous studies, in which significant decreasing or increasing trends in depressive symptoms over time period were found, especially among older adults (11, 56). Therefore, further observation of period-based trend on depression in a longer period may detect a more accurate picture.

We identified that urban–rural gaps in depression enlarged with age and then narrowed after 80, which was similar with the findings from Wu et al. (34). They used data from the China Family Panel Studies from 2016 to 2020, and also found that residence gaps in CES-D scores widen among younger samples, then narrowed among older samples. The increasing disparities in depression among younger samples was mainly due to a faster increase in depression among rural residents. The possible reason maybe that for these middle-aged and ‘young old’(younger than 80 years old), the urban residents could get better health services, retirement welfare, and various forms of support form their children and society than those in rural area (21). Meanwhile, some studies suggested that rural older adults in China had server disabilities and worse health status than urban older adults (57, 58), which were identified as risk factors of depression (47). The narrowed urban–rural disparities in depression on advanced age might be explained by the selective survival effect. Due to the urban–rural gaps in development level, those individuals who could survive to advanced age suffering possible hardships in rural area were likely to be more robust than peers live in urban area (36, 59). Those weaker individuals in rural with worse living conditions and health services could rarely reach old ages. Regarding cohort effects, a narrowing trend on urban–rural gaps in depression was detected. However, this trend was partly due to the upward trends in depressive symptoms of urban residents among younger cohorts, and partly due to the downward trends in depression among rural residents. Therefore, more attention should be given to younger cohorts in urban area to prevent or postpone the onset of depression and to ensure they obtain adequate care.

The present study has some limitations. Firstly, due to the limitations of the data, the earliest and latest birth cohorts may not fully represent the age distribution, which may bias the estimation for age and cohort trends. Future studies encompassing a longer period may be able to provide a more precise and comprehensive understanding of the age span of depressive changes. Secondly, we focused on basic effects of APC and individual variables on depression. Effects from social network, lifestyles, and life events could be further explored. Thirdly, CES-D was a self-reported retrospective indicator of depression, which may be susceptible to cognitive biases (21). Future studies could use clinician-administered scales to explore the APC effects on depression further.

In conclusion, this study provides an overview of age, period and cohort trends on depression and examine these three temporal effects on urban–rural residence disparities in depression among middle-aged and older Chinese adults. Given the observed stronger age effects on depression, coupled with the increasing depression trend among younger cohorts, the rapid ageing of the Chinese population will inevitably lead to an increase in the number of older adults with depression. In addition, the increase in depressive symptoms among urban younger cohorts also requires policy attention. Intervention issues should be taken to prevent or postpone the onset of depression.

## Data availability statement

Publicly available datasets were analyzed in this study. The datasets can be found here: https://charls.charlsdata.com/pages/data/111/en.html.

## Ethics statement

The studies involving humans were approved by Biomedical Ethics Review Committee of Peking University. The studies were conducted in accordance with the local legislation and institutional requirements. The participants provided their written informed consent to participate in this study.

## Author contributions

XH: Writing – original draft, Writing – review & editing. WJ: Writing – review & editing. JW: Writing – review & editing. HD: Writing – review & editing.

## References

[1] Institute of Health Metrics and Evaluation. GBD results. (2020). Available at: https://vizhub.healthdata.org/gbd-results/ (accessed January 4, 2024).

[2] Malhi GS Mann JJ. Depression. Lancet. (2018) 392:2299–312. doi: 10.1016/S0140-6736(18)31948-2 30396512

[3] World Health Organization. Depression and other common mental disorders. Geneva: Global Health Estimates (2017).

[4] Walker ER Mcgee RE Druss BG. Mortality in mental disorders and global disease burden implications: a systematic review and meta-analysis. JAMA Psychiatry. (2015) 72:334–41. doi: 10.1001/jamapsychiatry.2014.2502, PMID: 25671328 PMC4461039

[5] Haigh EAPP Bogucki OEM Sigmon STP Blazer DGMP. Depression among older adults: a 20-year update on five common myths and misconceptions. Am J Geriatr Psychiatry. (2018) 26:107–22. doi: 10.1016/j.jagp.2017.06.011, PMID: 28735658

[6] König H König H Konnopka A. The excess costs of depression: a systematic review and meta-analysis. Epidemiol Psychiatr Sci. (2019) 29:e30. doi: 10.1017/S2045796019000180, PMID: 30947759 PMC8061284

[7] Hu X Gu S Zhen X Sun X Gu Y Dong H. Trends in activities of daily living disability among Chinese older adults from 1998 to 2018: an age-period-cohort analysis. Eur J Ageing. (2022) 19:1167–79. doi: 10.1007/s10433-022-00690-6, PMID: 36506673 PMC9729626

[8] Yang Y Land KC. Age-period-cohort analysis: New models, methods, and empirical applications. Boca Raton: Taylor & Francis (2013).

[9] Handing EP Strobl C Jiao Y Feliciano L Aichele S. Predictors of depression among middle-aged and older men and women in Europe: a machine learning approach. Lancet Reg Health Eur. (2022) 18:100391. doi: 10.1016/j.lanepe.2022.100391, PMID: 35519235 PMC9065918

[10] Beller J Regidor E Lostao L Miething A Kröger C Safieddine B. Decline of depressive symptoms in Europe: differential trends across the lifespan. Soc Psychiatry Psychiatr Epidemiol. (2021) 56:1249–62. doi: 10.1007/s00127-020-01979-6, PMID: 33180149 PMC8225536

[11] Beller J. Age-period-cohort analysis of depression trends: are depressive symptoms increasing across generations in Germany? Eur J Ageing. (2022) 19:1493–505. doi: 10.1007/s10433-022-00732-z, PMID: 36506693 PMC9729517

[12] Bai R Dong W Peng Q Bai Z. Trends in depression incidence in China, 1990-2019. J Affect Disord. (2022) 296:291–7. doi: 10.1016/j.jad.2021.09.084, PMID: 34606800

[13] Pescosolido BA Halpern-Manners A Luo L Perry B. Trends in public stigma of mental illness in the US, 1996-2018. JAMA Netw Open. (2021) 4:e2140202. doi: 10.1001/jamanetworkopen.2021.40202 34932103 PMC8693212

[14] Stephan A Strobl R Schwettmann L Meisinger C Ladwig K Linkohr B. Being born in the aftermath of world war ii increases the risk for health deficit accumulation in older age: results from the kora-age study. Eur J Epidemiol. (2019) 34:675–87. doi: 10.1007/s10654-019-00515-4, PMID: 30941552

[15] Zhang P Lv Y Li Z Yin Z Li F Wang J. Age, period, and cohort effects on activities of daily living, physical performance, and cognitive functioning impairment among the oldest-old in China. J Gerontol A Biol Sci Med Sci. (2020) 75:1214–21. doi: 10.1093/gerona/glz196, PMID: 31435643 PMC7984417

[16] Huang C Phillips MR Zhang Y Zhang J Shi Q Song Z. Malnutrition in early life and adult mental health: evidence from a natural experiment. Soc Sci Med. (2013) 97:259–66. doi: 10.1016/j.socscimed.2012.09.051, PMID: 23313495 PMC3726543

[17] Lumey LH Stein AD Susser E. Prenatal famine and adult health. Annu Rev Public Health. (2011) 32:237–62. doi: 10.1146/annurev-publhealth-031210-101230, PMID: 21219171 PMC3857581

[18] Brown AS van Os J Driessens C Hoek HW Susser ES. Further evidence of relation between prenatal famine and major affective disorder. Am J Psychiatry. (2000) 157:190–5. doi: 10.1176/appi.ajp.157.2.190, PMID: 10671386

[19] Sullivan KJ Liu A Dodge HH Andreescu C Chang CH Ganguli M. Depression symptoms declining among older adults: birth cohort analyses from the rust belt. Am J Geriatr Psychiatry. (2020) 28:99–107. doi: 10.1016/j.jagp.2019.06.002, PMID: 31300193 PMC6898763

[20] Wild B Herzog W Schellberg D Lechner S Niehoff D Brenner H. Association between the prevalence of depression and age in a large representative German sample of people aged 53 to 80 years. Int J Geriatr Psychiatry. (2012) 27:375–81. doi: 10.1002/gps.2728, PMID: 21618284

[21] Zhang Y Zhao M. Gender disparities and depressive symptoms over the life course and across cohorts in China. J Affect Disord. (2021) 295:620–7. doi: 10.1016/j.jad.2021.08.134, PMID: 34509776

[22] Twenge JM. Time period and birth cohort differences in depressive symptoms in the u.s., 1982-2013. Soc Indic Res. (2015) 121:437–54. doi: 10.1007/s11205-014-0647-1

[23] Yang Y Land KC. Age-period-cohort analysis of repeated cross-section surveys: fixed or random effects? Sociol Methods Res. (2008) 36:297–326. doi: 10.1177/0049124106292360

[24] Yang G D’Arcy C. Age, period and cohort effects in depression prevalence among Canadians 65+, 1994 to 2018: a multi-level analysis. Int J Soc Psychiatry. (2023) 69:885–94. doi: 10.1177/00207640221141785, PMID: 36475530 PMC10248299

[25] He J Ouyang F Li L Qiu D Li Y Xiao S. Incidence trends of major depressive disorder in China: an age-period-cohort modeling study. J Affect Disord. (2021) 288:10–6. doi: 10.1016/j.jad.2021.03.075 33839553

[26] Gao M Kuang W Qiu P Wang H Lv X Yang M. The time trends of cognitive impairment incidence among older Chinese people in the community: based on the CLHLS cohorts from 1998 to 2014. Age Ageing. (2017) 46:787–93. doi: 10.1093/ageing/afx038, PMID: 28369164

[27] Peen J Schoevers RA Beekman AT Dekker J. The current status of urban-rural differences in psychiatric disorders. Acta Psychiatr Scand. (2010) 121:84–93. doi: 10.1111/j.1600-0447.2009.01438.x, PMID: 19624573

[28] Xin Y Ren X. Predicting depression among rural and urban disabled elderly in China using a random forest classifier. BMC Psychiatry. (2022) 22:118. doi: 10.1186/s12888-022-03742-4, PMID: 35168579 PMC8845343

[29] Purtle J Nelson KL Yang Y Langellier B Stankov I Diez Roux AV. Urban-rural differences in older adult depression: a systematic review and meta-analysis of comparative studies. Am J Prev Med. (2019) 56:603–13. doi: 10.1016/j.amepre.2018.11.008, PMID: 30777704

[30] Qin X Wang S Hsieh C. The prevalence of depression and depressive symptoms among adults in China: estimation based on a national household survey. China Econ Rev. (2018) 51:271–82. doi: 10.1016/j.chieco.2016.04.001

[31] Li L Liu J Xu H Zhang Z. Understanding rural-urban differences in depressive symptoms among older adults in China. J Aging Health. (2016) 28:341–62. doi: 10.1177/0898264315591003, PMID: 26100620 PMC4893784

[32] Chiao C Weng L Botticello AL. Social participation reduces depressive symptoms among older adults: an 18-year longitudinal analysis in Taiwan. BMC Public Health. (2011) 11:292. doi: 10.1186/1471-2458-11-292, PMID: 21569285 PMC3103460

[33] Li L Wu C Gan Y Qu X Lu Z. Insomnia and the risk of depression: a meta-analysis of prospective cohort studies. BMC Psychiatry. (2016) 16:375. doi: 10.1186/s12888-016-1075-3, PMID: 27816065 PMC5097837

[34] Wu Y Su B Chen C Zhao Y Zhong P Zheng X. Urban-rural disparities in the prevalence and trends of depressive symptoms among Chinese elderly and their associated factors. J Affect Disord. (2023) 340:258–68. doi: 10.1016/j.jad.2023.07.117, PMID: 37536424

[35] Zhao Y Hu Y Smith JP Strauss J Yang G. Cohort profile: the China health and retirement longitudinal study (CHARLS). Int J Epidemiol. (2014) 43:61–8. doi: 10.1093/ije/dys203, PMID: 23243115 PMC3937970

[36] Zeng Y Vaupel JW Xiao Z Zhang C Liu Y. Sociodemographic and health profiles of the oldest old in China. Popul Dev Rev. (2002) 28:251–73. doi: 10.1111/j.1728-4457.2002.00251.x

[37] Radloff LS. The ces-d scale: a self-report depression scale for research in the general population. Appl Psychol Meas. (1977) 1:385–401. doi: 10.1177/014662167700100306

[38] Kliem S Beller J Tibubos AN Zenger M Schmalbach B Brähler E. A reanalysis of the center for epidemiological studies depression scale (ces-d) using non-parametric item response theory. Psychiatry Res. (2020) 290:113132. doi: 10.1016/j.psychres.2020.113132, PMID: 32521379

[39] Chen H Mui AC. Factorial validity of the center for epidemiologic studies depression scale short form in older population in China. Int Psychogeriatr. (2014) 26:49–57. doi: 10.1017/S1041610213001701, PMID: 24125553

[40] van de Rest O van der Zwaluw N Beekman AT de Groot LC Geleijnse JM. The reliability of three depression rating scales in a general population of dutch older persons. Int J Geriatr Psychiatry. (2010) 25:998–1005. doi: 10.1002/gps.2449, PMID: 19998315

[41] Lei X Sun X Strauss J Zhang P Zhao Y. Depressive symptoms and SES among the mid-aged and elderly in China: evidence from the China health and retirement longitudinal study national baseline. Soc Sci Med. (2014) 120:224–32. doi: 10.1016/j.socscimed.2014.09.028, PMID: 25261616 PMC4337774

[42] Lin S Beck AN Finch BK Hummer RA Master RK. Trends in US older adult disability: exploring age, period, and cohort effects. Am J Public Health. (2012) 102:2157–63. doi: 10.2105/AJPH.2011.300602, PMID: 22994192 PMC3471673

[43] Chen F Yang Y Liu G. Social change and socioeconomic disparities in health over the life course in China: a cohort analysis. Am Sociol Rev. (2010) 75:126–50. doi: 10.1177/0003122409359165, PMID: 20379373 PMC2850448

[44] Yang Y. Social inequalities in happiness in the United States, 1972 to 2004: an age-period-cohort analysis. Am Sociol Rev. (2008) 73:204–26. doi: 10.1177/000312240807300202

[45] Zhang L. An age-period-cohort analysis of religious involvement and adult self-rated health: results from the USA, 1972-2008. J Relig Health. (2017) 56:916–45. doi: 10.1007/s10943-016-0292-x, PMID: 27464644

[46] Hu X Gu S Zhen X Sun X Gu Y Dong H. Trends in cognitive function among Chinese elderly from 1998 to 2018: an age-period-cohort analysis. Front Public Health. (2021) 9:753671. doi: 10.3389/fpubh.2021.753671, PMID: 34900900 PMC8660074

[47] Blazer D. Depression in late life: review and commentary. J Gerontol A Biol Sci Med Sci. (2003) 58:249–65. doi: 10.1093/gerona/58.3.m249, PMID: 12634292

[48] Feng Z Jones K Phillips DR. Social exclusion, self-rated health and depression among older people in China: evidence from a national survey of older persons. Arch Gerontol Geriatr. (2019) 82:238–44. doi: 10.1016/j.archger.2019.02.016, PMID: 30875525

[49] Sutin AR Terracciano A Milaneschi Y An Y Ferrucci L Zonderman AB. The trajectory of depressive symptoms across the adult life span. JAMA Psychiatry. (2013) 70:803–11. doi: 10.1001/jamapsychiatry.2013.193, PMID: 23760442 PMC3740038

[50] Danner DD Snowdon DA Friesen WV. Positive emotions in early life and longevity: findings from the nun study. J Pers Soc Psychol. (2001) 80:804–13. doi: 10.1037/0022-3514.80.5.804, PMID: 11374751

[51] Guo S Zheng XY. New evidence of trends in cognitive function among middle-aged and older adults in China, 2011-2018: an age-period-cohort analysis. BMC Geriatr. (2023) 23:498. doi: 10.1186/s12877-023-04166-9 37605117 PMC10440902

[52] Xie P Wu K Zheng Y Guo Y Yang Y He J. Prevalence of childhood trauma and correlations between childhood trauma, suicidal ideation, and social support in patients with depression, bipolar disorder, and schizophrenia in southern China. J Affect Disord. (2018) 228:41–8. doi: 10.1016/j.jad.2017.11.011, PMID: 29223913

[53] Zhang Z Gu D Hayward M. Early life influences on cognitive impairment among oldest old Chinese. J Gerontol B Psychol Sci Soc Sci. (2008) 63:S25–33. doi: 10.1093/geronb/63.1.s25, PMID: 18332198

[54] Eckersley R Dear K. Cultural correlates of youth suicide. Soc Sci Med. (2002) 55:1891–904. doi: 10.1016/S0277-9536(01)00319-7 12406459

[55] Deborah D. Urban consumer culture. The. China Q. (2005) 183:692–709. doi: 10.1017/S0305741005000421

[56] Yan X Lin S Li J Wei Y Pei L. Temporal trends in the incidence of depressive disorders across China, Japan, and South Korea: an age-period-cohort analysis for the global burden of disease study 2019. Int J Ment Health Addict. (2023). doi: 10.1007/s11469-023-01220-w

[57] Zimmer Z Wen M Kaneda T. A multi-level analysis of urban/rural and socioeconomic differences in functional health status transition among older Chinese. Soc Sci Med. (2010) 71:559–67. doi: 10.1016/j.socscimed.2010.03.048, PMID: 20621749 PMC2904335

[58] Yu P Song X Shi J Mitnitski A Tang Z Fang X. Frailty and survival of older Chinese adults in urban and rural areas: results from the Beijing longitudinal study of aging. Arch Gerontol Geriatr. (2012) 54:3–8. doi: 10.1016/j.archger.2011.04.020, PMID: 21621282

[59] Zimmer Z Martin LG Nagin DS Jones BL. Modeling disability trajectories and mortality of the oldest-old in China. Demography. (2012) 49:291–314. doi: 10.1007/s13524-011-0075-7, PMID: 22246796

