# Supplementary material for: Age, period, cohort effects in trends of depressive symptoms among middle-aged and older Chinese adults
Source: Front Public Health. 2024 Jul 31;12:1383512. doi: 10.3389/fpubh.2024.1383512 (PMC11321982; doi:10.3389/fpubh.2024.1383512)
Supplement: Supplementary file 1 [file Data_Sheet_1.docx]

Supplementary Material

# Supplementary Table 1. CES-D-10 questions

| **Number** | **CES-D items** |
| --- | --- |
| 1 | I was bothered by things that don't usually bother me. |
| 2 | I had trouble keeping my mind on what I was doing. |
| 3 | I felt depressed. |
| 4 | I felt everything I did was an effort. |
| 5 | I felt hopeful about the future. |
| 6 | I felt fearful. |
| 7 | My sleep was restless. |
| 8 | I was happy. |
| 9 | I felt lonely. |
| 10 | I could not get “going.” |


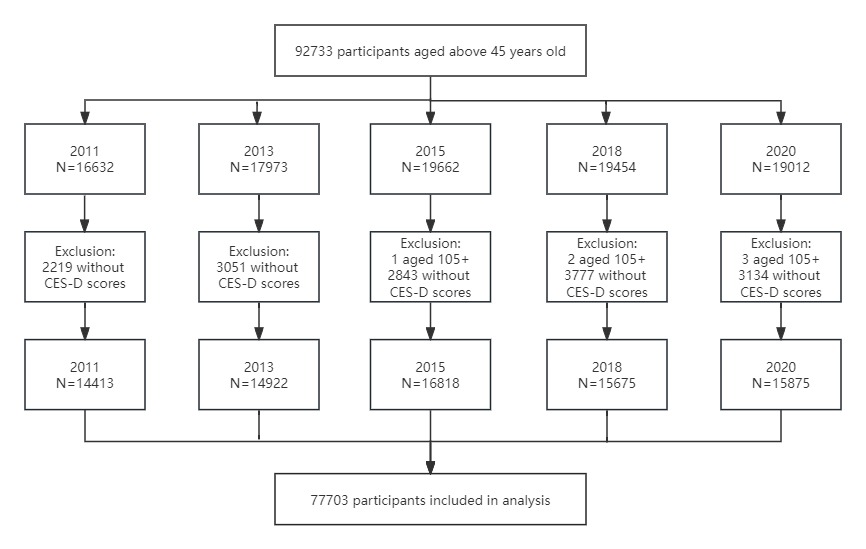


**Supplementary Figure 1.** Flowchart of the study sample

**Sensitivity analyses**

In order to reduce the potential bias of excluding the missing data, we performed multiple imputation on CES-D scores and the analytic sample was expanded to 92727 observations. As shown in Supplementary Table 2-3 and Supplementary Figure 2-3, estimated results from CCREMs and predicted APC effects were similar compared to the estimates in the main analysis in Table 1-2 and Figure 1-2 in the main text.

**Supplementary Table 2. Basic characteristics of samples in five surveys after imputation**

| Variables | ALL(N=92727) | 2011(N=16632) | 2013(N=17973) | 2015(N=19661) | 2018(N=19452) | 2020(N=19009) |
| --- | --- | --- | --- | --- | --- | --- |
| Age | 60.88±10.09 | 59.08±9.76 | 59.93±9.93 | 60.19±10.13 | 61.74±10.15 | 63.16±9.91 |
| Sex |  |  |  |  |  |  |
| male | 44675(48.2) | 8125(48.9) | 8731(48.6) | 9573(48.7) | 9282(47.7) | 8964(47.2) |
| female | 48052(51.8) | 8507(51.1) | 9242(51.4) | 10088(51.3) | 10170(52.3) | 10045(52.8) |
| Residence |  |  |  |  |  |  |
| urban | 37336(40.3) | 6659(40.0) | 7254(40.4) | 7989(40.6) | 7832(40.3) | 7602(40.0) |
| rural | 55391(59.7) | 9973(60.0) | 10719(59.6) | 11672(59.4) | 11620(59.7) | 11407(60.0) |
| Co-residence |  |  |  |  |  |  |
| alone | 6341(6.8) | 982(5.9) | 889(4.9) | 1042(5.3) | 1771(9.1) | 1657(8.7) |
| with others | 86386(93.2) | 15650(94.1) | 17084(95.1) | 18619(94.7) | 17681(90.9) | 17352(91.3) |
| Marital status |  |  |  |  |  |  |
| with spouse | 79601(85.8) | 14480(87.1) | 15618(86.9) | 16993(86.4) | 16549(85.1) | 15961(84.0) |
| without spouse | 13126(14.2) | 2152(12.9) | 2355(13.1) | 2668(13.6) | 2903(14.9) | 3048(16.0) |
| Education |  |  |  |  |  |  |
| illiterate | 23464(25.3) | 4573(27.5) | 4736(26.4) | 4947(25.2) | 4713(24.2) | 4495(23.6) |
| primary | 39613(42.7) | 6512(39.2) | 7087(39.4) | 8625(43.9) | 8814(45.3) | 8575(45.1) |
| secondary or above | 29638(32.0) | 5543(33.3) | 6148(34.2) | 6083(30.9) | 5925(30.5) | 5939(31.2) |
| missing^ | 12 | 4(＜0.1) | 2(＜0.1) | 6(＜0.1) | - | - |
| Working status |  |  |  |  |  |  |
| not currently working | 32626(35.8) | 6050(37.3) | 6080(34.8) | 6653(34.8) | 7057(36.5) | 6786(35.8) |
| currently working | 58523(64.2) | 10178(62.7) | 11395(65.2) | 12460(65.2) | 12301(63.5) | 12189(64.2) |
| missing | 1578(1.7) | 404(2.4) | 498(2.8) | 548(2.9) | 94(0.5) | 34(0.2) |
| ADL limitations | 0.41±1.07 | 0.37±1.02 | 0.36±0.98 | 0.42±1.06 | 0.42±1.10 | 0.49±1.16 |
| CES-D score | 8.25±6.16 | 8.44±6.22 | 7.89±5.71 | 7.90±6.27 | 8.41±6.33 | 8.62±6.19 |

*Note*. CES-D = the Center for Epidemiologic Studies Depression Scale. ADL = activities of daily living.

Data are presented as mean± standard deviation or n (%).

^ Missing data were excluded from other percentage calculation.

**Supplementary Table 3. Hierarchical age-period-cohort cross-classified random-effects model estimates of CES-D scores after imputation**

| Fixed Effects | Model 1 | | Model 2 | | Model 3 | | Model 4 | | Model 5 | | Model 6 | |
| --- | --- | --- | --- | --- | --- | --- | --- | --- | --- | --- | --- | --- |
|  | Coefficient | SE | Coefficient | SE | Coefficient | SE | Coefficient | SE | Coefficient | SE | Coefficient | SE |
| Intercept | 8.059 ^***^ | 0.139 | 7.106 ^***^ | 0.137 | 7.002^***^ | 0.139 | 6.890 ^***^ | 0.176 | 6.962 ^***^ | 0.155 | 6.892 ^***^ | 0.183 |
| Age | 0.528 ^***^ | 0.052 | 0.510 ^***^ | 0.042 | 0.424 ^***^ | 0.048 | -0.115^*^ | 0.058 | 0.439 ^***^ | 0.069 | -0.118 | 0.068 |
| Age^2^ | -0.142 ^***^ | 0.023 | -0.146 ^***^ | 0.021 | -0.052 ^*^ | 0.026 | -0.252 ^***^ | 0.027 | -0.031 | 0.031 | -0.253 ^***^ | 0.030 |
| Residence (urban = 0) |  |  | 1.930 ^***^ | 0.040 | 2.125 ^***^ | 0.052 | 1.527 ^***^ | 0.053 | 2.181 ^***^ | 0.131 | 1.521 ^***^ | 0.112 |
| Age * Residence |  |  |  |  | 0.175 ^***^ | 0.043 | 0.058 | 0.043 |  |  | 0.019 | 0.072 |
| Age^2^ * Residence |  |  |  |  | -0.182 ^***^ | 0.030 | -0.130 ^***^ | 0.030 |  |  | -0.110^**^ | 0.040 |
| Sex (male = 0) |  |  |  |  |  |  | 1.329 ^***^ | 0.042 |  |  | 1.327 ^***^ | 0.042 |
| Education level (illiterate = 0) |  |  |  |  |  |  |  |  |  |  |  |  |
| primary |  |  |  |  |  |  | -0.315 ^***^ | 0.054 |  |  | -0.318 ^***^ | 0.054 |
| secondary or above |  |  |  |  |  |  | -1.409 ^***^ | 0.060 |  |  | -1.411^***^ | 0.060 |
| Marriage (have spouse = 0) |  |  |  |  |  |  | 1.122 ^***^ | 0.069 |  |  | 1.123^***^ | 0.069 |
| Co-residence (alone = 0) |  |  |  |  |  |  | -0.061 | 0.091 |  |  | -0.064 | 0.091 |
| Working status (no = 0) |  |  |  |  |  |  | -0.067 | 0.047 |  |  | -0.068 | 0.047 |
| ADL limitations |  |  |  |  |  |  | 1.363 ^***^ | 0.019 |  |  | 1.363 ^***^ | 0.019 |
| Variance Components | Model 1 | | Model 2 | | Model 3 | | Model 4 | | Model 5 | | Model 6 | |
|  | Coefficient | SE | Coefficient | SE | Coefficient | SE | Coefficient | SE | Coefficient | SE | Coefficient | SE |
| Period |  |  |  |  |  |  |  |  |  |  |  |  |
| Intercept | 0.078 | 0.057 | 0.080 | 0.058 | 0.080 | 0.058 | 0.071 | 0.051 | 0.083 | 0.061 | 0.072 | 0.054 |
| Residence |  |  |  |  |  |  |  |  | 0.027 | 0.025 | 0.023 | 0.022 |
| Cohort |  |  |  |  |  |  |  |  |  |  |  |  |
| Intercept | 0.033 ^*^ | 0.018 | 0.018 ^*^ | 0.011 | 0.019 ^*^ | 0.012 | 0.034^*^ | 0.018 | 0.063 ^*^ | 0.034 | 0.059 ^*^ | 0.032 |
| Residence |  |  |  |  |  |  |  |  | 0.094 ^*^ | 0.053 | 0.050 | 0.032 |
| Model Fit |  |  |  |  |  |  |  |  |  |  |  |  |
| BIC | 583028.5 | | 580707.6 | | 580679.7 | | 556280.0 | | 580643.6 | | 556255.1 | |

*Note*. CES-D = the Center for Epidemiologic Studies Depression Scale. SE = standard error. ADL = activities of daily living. BIC = Bayesian Information Criterion.

* p ≤ 0.05;** p ≤ 0.01;*** p ≤ 0.001

**A**

**B**

**C**

**Supplementary Figure 2. Overall age, period, and cohort effects on CES-D scores after imputation (A) Age (B) Cohort (C) Period**

**A**

**B**

**C**

**Supplementary Figure 3. Predicted age, period, and cohort trends in the urban-rural disparity in CES-D scores after imputation (A) Age (B) Cohort (C) Period**
